# Leukocyte-Derived Extracellular Vesicles in Blood with and without EpCAM Enrichment

**DOI:** 10.3390/cells8080937

**Published:** 2019-08-20

**Authors:** Afroditi Nanou, Leonie L. Zeune, Leon W.M.M. Terstappen

**Affiliations:** Department of Medical Cell BioPhysics, Faculty of Science and Technology, University of Twente, 7522 NH Enschede, The Netherlands

**Keywords:** leukocyte-derived extracellular vesicles, immunofluorescence imaging, EpCAM enrichment, CellSearch, EasyCount slides, ACCEPT

## Abstract

Large tumor-derived Extracellular Vesicles (tdEVs) detected in blood of metastatic prostate, breast, colorectal, and non-small cell lung cancer patients after enrichment for Epithelial Cell Adhesion Molecule (EpCAM) expression and labeling with 4′,6-diamidino-2-phenylindole (DAPI), phycoerythrin-conjugated antibodies against Cytokeratins (CK-PE), and allophycocyanin-conjugated antibody against the cluster of differentiation 45 (CD45-APC), are negatively associated with the overall survival of patients. Here, we investigated whether, similarly to tdEVs, leukocyte-derived EVs (ldEVs) could also be detected in EpCAM-enriched blood. Presence of ldEVs and leukocytes in image data sets of EpCAM-enriched samples of 25 healthy individuals and 75 metastatic cancer patients was evaluated using the ACCEPT software. Large ldEVs could indeed be detected, but in contrast to the 20-fold higher frequency of tdEVs as compared to Circulating Tumor Cells (CTCs), ldEVs were present in a 5-fold lower frequency as compared to leukocytes. To evaluate whether these ldEVs pre-exist in the blood or are formed during the CellSearch procedure, the blood of healthy individuals without EpCAM enrichment was labelled with the nuclear dye Hoechst and fluorescently tagged monoclonal antibodies recognizing the leukocyte-specific CD45, platelet-specific CD61, and red blood cell-specific CD235a. Fluorescence microscopy imaging using a similar setup as the CellSearch was performed and demonstrated the presence of a similar population of ldEVs present at a 3-fold lower frequency as compared to leukocytes.

## 1. Introduction

During the last decades, Extracellular Vesicles (EVs) have emerged as promising disease biomarkers bearing similar membrane and cargo composition as their originating cells [1,2,3]. Importantly, for nucleic acid analysis, the membrane encapsulated nucleic acid cargo is protected from enzymatic degradation, and consequently, it can circulate for a longer time compared to cell-free DNA (cfDNA) [4,5]. In the case of cancer, the presence of nucleic acids (DNA, mRNA, and miRNA) within tumor-derived EVs (tdEVs) and proteins within or on tdEV membranes could provide information of the predisposition of the tumor to metastasize in specific organs and guide treatment monitoring of patients to block metastasis and cancer progression [5,6,7,8,9]. It has been demonstrated that EVs in biofluids of cancer patients are significantly elevated when compared to the respective numbers of healthy individuals [10,11]. However, to our knowledge, there is no data available in regards to the composition of the redundant EVs in the blood of cancer patients. The recent *in vivo* studies of Ricklefs et al. using brain tumors expressing the green fluorescent protein (GFP) in mice showed that less than 0.5% of the total circulating EVs were GFP+ [10]. That finding implies that more cell types secrete EVs in response to the present tumor contributing to the final EV pool detected in biofluids of cancer patients. Furthermore, the pre-analytical steps of sample processing determine the EV populations to be analyzed and could lead to biased conclusions. The majority of research groups is only interested in exosomes that constitute the smallest subclass of EVs as they consider them products of active cell secretion; therefore, they are using differential centrifugation steps to get rid of other EV subclasses, collect the exosome fraction as a pellet from the final ultracentrifugation step and label them with antibodies recognizing generic exosome-enriched biomarkers, mainly tetraspanins, such as the clusters of differentiation CD81, CD9, and CD63 to identify them [10,12]. Nevertheless, EV subclasses of larger size (microvesicles, oncosomes, and apoptotic bodies) have been reported to be bioactive with a wide spectrum of functions depending on their cells of origin [13]. Importantly, Vagner et al. reported the presence of DNA in large tdEVs reflecting the genetic aberrations of the tumor; a finding that highlights their promising potential in the liquid biopsy field [9]. Padda et al. also demonstrated that the majority of prostate-specific membrane antigen (PSMA) expressing EVs in plasma of prostate cancer patients derive directly from the plasma membrane and have a larger size [14]; hence, these clinically important populations are missed by solely the exosome analysis. Very few studies have investigated the isolation and downstream characterization of specific tdEVs from patient samples using immuno-affinity techniques [15,16]. Recently, we showed that large tdEVs, immunomagnetically isolated based on their EpCAM expression together with Circulating Tumor Cells (CTCs) by the CellSearch system from the blood of metastatic prostate, breast, colorectal, and non-small cell lung cancer patients have equivalent prognostic power to CTCs [16,17]. These observations were enabled through the availability of the open-source ACCEPT image analysis program, which allows for the exploration and enumeration in a single level of all different classes of objects detected in the fluorescence images in an automated, fast and reproducible manner, free of the subjectivity and bias of different operators [18,19]. However, it is not clear whether our previously reported large tdEVs are a result of the fragmentation of CTCs during the immunomagnetic EpCAM enrichment and washing steps that the CellSearch system is using or whether they pre-exist in the blood samples of cancer patients. Their rare frequency in combination with the abundance of blood cells and EVs of different origins prevent us from addressing that question by labeling of blood samples without any pre-enrichment steps and subsequent enumeration from fluorescence images. In this study, we identified in the digitally stored CellSearch images some CD45+, DAPI-, CK- objects of similar size to tdEVs that we baptized leukocyte-derived Extracellular Vesicles (ldEVs). We addressed the question of whether large ldEVs pre-exist in the blood of individuals without EpCAM enrichment or they are by-products of cell fragmentation by the CellSearch procedure. Towards that direction, we labeled blood samples of healthy individuals with the nuclear dye Hoechst and fluorophore-conjugated antibodies against the leukocyte-specific CD45, the platelet-specific CD61, and the red blood cell-specific CD235a without any pre-enrichment or pre-analytical steps. The samples were imaged using a fluorescence microscope with a 10×/0.45 numerical aperture (NA) objective to enable fair comparison of the image datasets acquired by the CellTracks Analyzer II of the CellSearch system [20].

## 2. Materials and Methods

### 2.1. Immunofluorescence Image Data Sets of EpCAM-Enriched Cells and Extracellular Vesicles of 25 Healthy Individuals and 75 Metastatic Cancer Patients

One-hundred digitally stored CellSearch image data sets corresponding to EpCAM-enriched blood samples of 25 healthy individuals, 25 metastatic prostate (CRPC), 25 colorectal (mCRC), and 25 non-small cell lung (NSCLC) cancer patients before the initiation of a new therapy, were used for this analysis. The EpCAM-enriched leukocytes and large leukocyte-derived EVs present in these fluorescence images were enumerated. The CRPC and mCRC patients had participated in the retrospective IMMC38 (***NCT00133900***) and CAIRO 2 (***NCT00208546***) clinical studies, respectively.

Briefly, the EpCAM+ Circulating Tumor Cells (CTCs) and tdEVs were positively selected by ferrofluids conjugated to an antibody recognizing the extracellular epitope of Epithelial Cell Adhesion Molecule (EpCAM, clone VU1D9) from 7.5 mL of the blood of cancer patients using the CellSearch system (Menarini Silicon Biosystems, Huntingdon Valley PA, USA), as previously described [21]. Following EpCAM immunomagnetic enrichment, the suspension was incubated with the nuclear dye DAPI and antibodies against the epithelial-specific cytokeratins 8, 18, and 19 (clone C11) conjugated to phycoerythrin (PE) and an antibody against the leukocyte-specific cluster of differentiation CD45 conjugated to allophycocyanin (APC). The suspension was transferred to a cartridge placed within a magnest that allowed for a homogeneous distribution of the ferrofluids and the EpCAM-enriched objects on a focal plane [22]. The cartridges were imaged using a semi-automated 10×/0.45 NA objective fluorescence microscope, the CellTracks Analyzer II, as previously described [21].

### 2.2. Blood Samples of 10 Healthy Individuals

Blood samples from 10 anonymous healthy individuals were collected in ethylenediaminetetraacetic acid (EDTA) tubes after written informed consent from the Experimental Centre for Technical Medicine (ECTM) donor service (University of Twente, Enschede, The Netherlands). The frequencies of white blood cells, red blood cells, and platelets were assessed using a hematology analyzer (Beckman Coulter, Brea, CA, USA). The samples were processed on the same day of the drawing.

### 2.3. Immunofluorescence Imaging of Cells and Extracellular Vesicles in Whole Blood Samples

10–20 μL of EDTA blood samples of 10 different healthy individuals were 10× diluted in 0.2 μm filtered 1% *w*/*v* bovine serum albumin (BSA) in phosphate- buffered saline (PBS) solution. Blood was incubated with the nuclear dye Hoechst 33342 (Invitrogen, cat. # H3570), the fluorescently tagged monoclonal antibodies CD45-PerCP (clone HI30 Invitrogen, cat. # MHCD4531) recognizing leukocytes, CD235a-Alexa Fluor^®^ 647 (clone YTH89.1, bio-rad, MCA 506A647) antibodies recognizing erythrocytes and CD61-Alexa Fluor^®^ 488 (clone Y2.51, bio-rad, cat. # MCA 2588A488) antibodies recognizing platelets. The final concentrations used were 4.0 μg/mL Hoechst, 0.5 μg/mL CD45-PerCP, 2.5 μg/mL CD235a-Alexa 647, and 0.6 μg/mL CD61-Alexa 488. The samples were incubated with the antibodies at 37 °C for 1–2 h and stored at 4 °C until further processing. Subsequently, the samples were further diluted to a final dilution of 500×. 10 μL of the diluted sample (corresponding to 0.02 μL of undiluted blood) were loaded in a well of EasyCount^TM^ Slide-6^TM^ (Immunicon Corp., Huntingdon Valley, PA, USA). Four–six technical replicates of samples were used to assess the reproducibility of the measurements. Image data sets of 55–65 frames/channel were acquired to cover the whole surface of each well using a semi-automated inverted fluorescence microscope (Eclipse Ti-E, Nikon Instruments, Amsterdam, The Netherlands) equipped with a 10×/0.45 numerical aperture (NA) objective, a camera (Orca flash 4.0 LT, C11440, Hamamatsu, Almere, The Netherlands) and fluorescence filter cubes (DAPI, FITC, PerCP, APC filter sets for the detection of Hoechst, CD61-Alexa 488, CD45-PerCP and CD235a-Alexa 647, respectively). The operator determined three corners of the surface to be scanned and adjusted the focus at four points distributed throughout each well. The exposure times used for the imaging were 20 ms for DAPI, 400 ms for PerCP, 500 ms for FITC, 1000 ms for APC, and 500 ms for brightfield. Few images were obtained using a 60×/0.70 NA objective and the same exposure times for comparison. However, only the images obtained with the 10× objective were used as an input for the enumeration of the different populations to allow a fair comparison with the images of the CellTracks Analyzer II. An example of a frame acquired with the 10× and 60× objectives is shown in Supplementary Figure S1.

### 2.4. Automated Enumeration of Objects in Immunofluorescence Images Using the Open-Source ACCEPT Software

All immunofluorescence image data sets, obtained with the 10×/0.45 NA objective microscopes, were processed with the open-source software for Automated CTC Classification, Enumeration and Phenotyping (ACCEPT) (http://github.com/LeonieZ/ACCEPT). The software detects all present objects, larger than four pixels, and extracts for each of them 10 morphological and fluorescence signal intensity measurements per fluorescence channel [18]. The user can design linear gates based on these features to define the classes of their interest and enumerate the objects falling within them [16,23]. The application of the same gates for all different samples allows the elimination of inter- and intra- operator variations leading subsequently, to a more objective consensus [24].

For the CellSearch generated images, gates for the enumeration of leukocyte-derived Extracellular Vesicles (ldEVs) and leukocytes were applied. The gates are summarized in Table 1. For the image data sets corresponding to the EasyCount Slides-6, gates for the enumeration of red blood cells, leukocytes, platelets and ldEVs were used and are summarized in Table 1.

## 3. Results

### 3.1. Detection of ldEVs in EpCAM-Enriched Blood Samples of Healthy Individuals and Metastatic Cancer Patients

**Figure 1:**
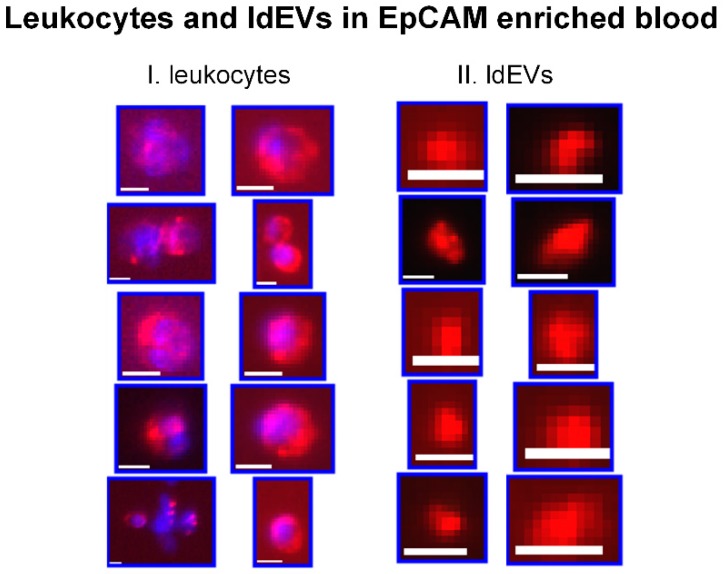
Thumbnails of I. leukocytes and II. Leukocyte-derived Extracellular Vesicles (ldEVs) detected in EpCAM-enriched blood samples. The red color represents CD45 and blue represents DAPI. Scale bars indicate 6.4 μm.

After careful examination of the immunofluorescence images of the CellSearch cartridges, CD45+, DAPI-, CK- objects, that resemble in size our previously reported CD45-, DAPI-, CK+ tdEVs [16,25], could be observed. We baptized these objects leukocyte-derived Extracellular Vesicles (ldEVs). Examples of single ldEV events in EpCAM-enriched samples by the CellSearch system are shown in Figure 1 next to some examples of leukocytes as a reference to their size and CD45 phenotype. The observation of the presence of these ldEVs in the CellSearch cartridges raised questions about their formation: are they fragments of leukocytes formed during the CellSearch procedure or do they pre-exist in the blood circulation?

### 3.2. Detection of Cell and Extracellular Vesicle Classes in the Blood Of Healthy Individuals without EpCAM Enrichment

**Figure 2:**
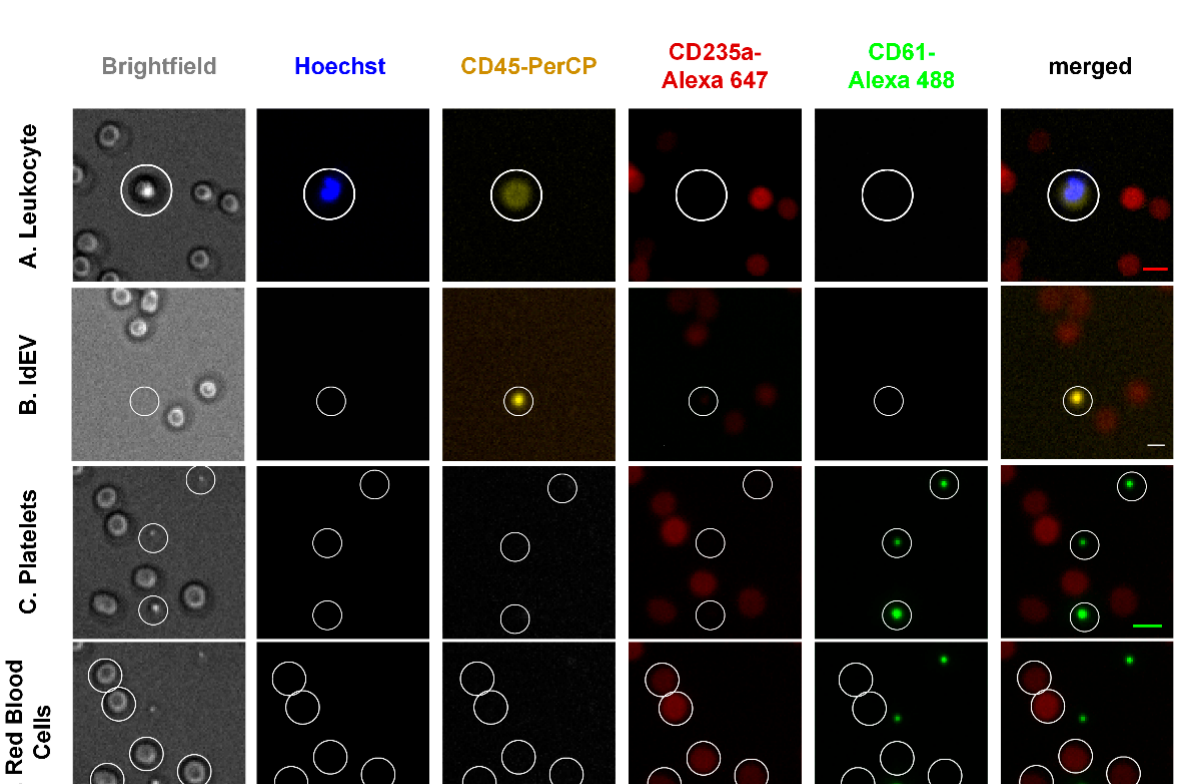
Bright field and immunofluorescence (IF) images of leukocytes, ldEVs, platelets and red blood cells in blood samples without EpCAM enrichment. Scale bars in the merged IF images indicate 10 μm.

In order to address the aforementioned question, blood samples of healthy individuals were labeled with Hoechst, CD45-PerCP, CD61-Alexa 488, and CD235a-Alexa 647 and were imaged with a similar fluorescence microscope as the CellTracks Analyzer II. No pre-enrichment or washing steps were used in order to minimize the cell fragmentation or activation. The inclusion of the aforementioned antibodies allowed the detection of four different classes of objects in the whole blood of healthy individuals, namely leukocytes, platelets, red blood cells, and ldEVs, as shown in Figure 2. Leukocytes are defined as nucleated CD45+, CD61-, CD235a- cells of a size between 7 and 20 μm (Panel A); leukocyte-derived Extracellular Vesicles (ldEVs) as CD45+, CD61-, CD235- objects without a nucleus and of undefined size as shown in the respective brightfield image (Panel B); platelets as CD45-, CD61+, CD235a- objects without a nucleus of size between 2 and 5 μm (Panel C) and red blood cells as CD45-, CD61-, CD235a+ cells without a nucleus and a size range between 6 and 10 μm (Panel D). In Panel C, three platelets are shown, of which the middle one is clearly smaller and with a lower expression of CD61; examination at higher magnification would allow for better identification of the smaller size platelets, but no discrimination could be made between small platelets and larger platelet-derived EVs. The presence of ldEVs in blood samples without pre-enrichment (Panel B) confirmed their pre-existence in whole blood.

### 3.3. ACCEPT Gates for the Automated Enumeration of Different Classes in the Blood with and without EpCAM Enrichment.

**Figure 3:**
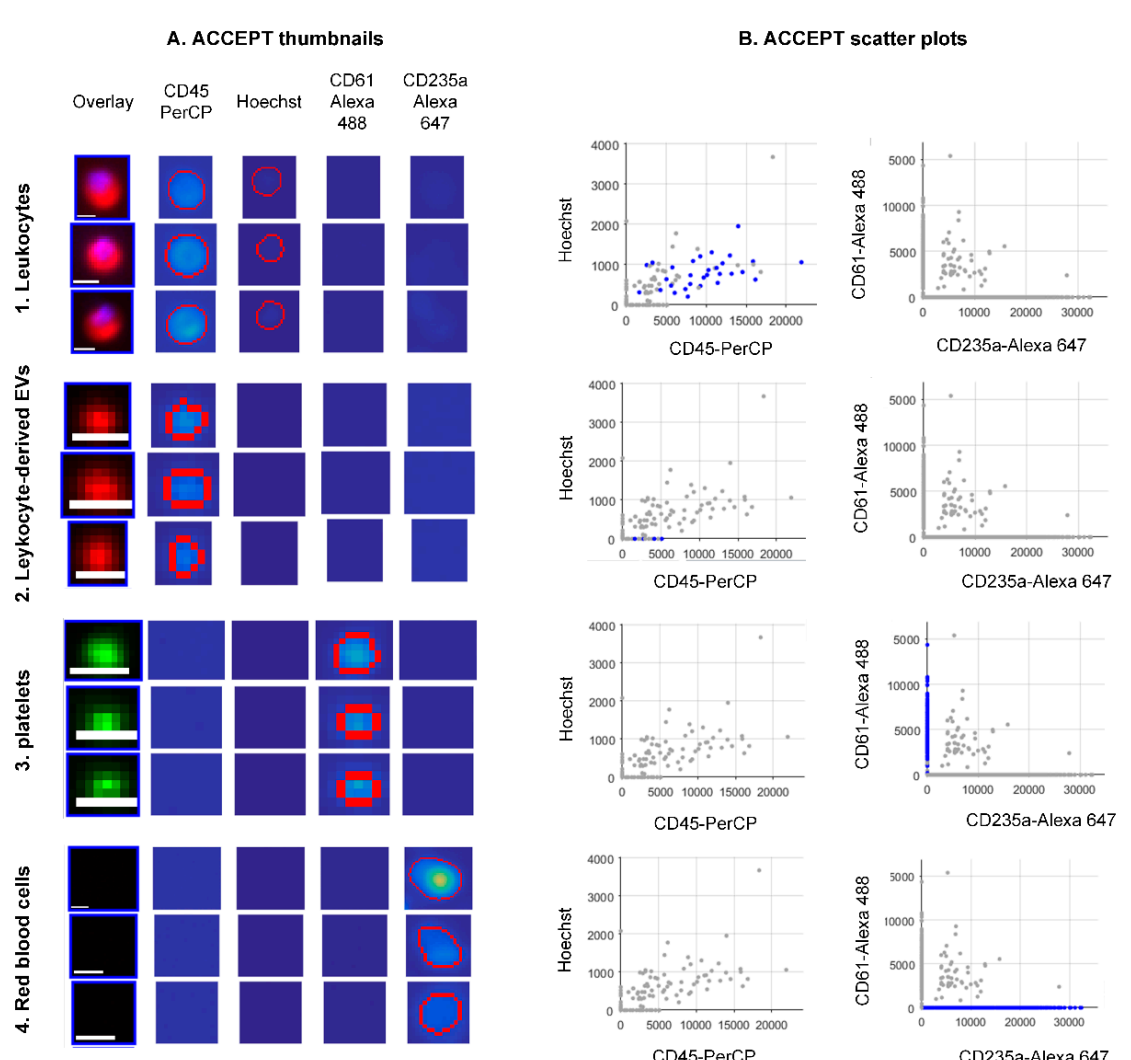
Examples of objects detected in the immunofluorescence image data sets of blood samples without EpCAM enrichment. The objects fall in the ACCEPT gates, the names of which are indicated vertically. Panel (**A**) shows examples of ACCEPT thumbnails. Scale bars indicate 6.4 μm. Panel (**B**) shows scatter plots of the mean intensity of the Hoechst versus the mean intensity of CD45-PerCP and the mean intensity of CD61-Alexa 488 versus the mean intensity of CD235a-Alexa 647. Blue dots represent single events falling in the respective gate.

In order to acquire the absolute counts of the different classes from each data set of the healthy individuals in a fast and unbiased manner, we processed all data sets with the open-source ACCEPT software. Based on the aforementioned characteristics of the different classes, we developed linear gates to enumerate the objects falling within each class automatically. The gates are summarized in Table 1. Three examples of objects per class are shown in Figure 3 (Panel A). The objects that fall into each class are depicted in blue dots in the scatter plots (Panel B) showing the mean Hoechst intensity versus the mean CD45-PerCP intensity and the mean CD61-Alexa 488 intensity versus the mean CD235a-Alexa 647 intensity. Objects falling in the “leukocyte” gate are double-positive for CD45 and Hoechst and negative for CD61 and CD235a, (Panel B1); ldEVs are positive only for CD45 (Panel B2); platelets are only positive for CD61 (Panel B3) and red blood cells are only positive for CD235a (Panel B4).

In order to achieve a fair comparison between the leukocyte and ldEV counts detected in the blood with and without EpCAM enrichment, very similar ACCEPT gates were developed for the automated enumeration of the two classes applied in the different image data sets. The gates can be found in Table 1.

The size threshold of 20 × 20 μm^2^ in the case of the “leukocyte” gate that was applied in blood samples with no enrichment was removed in the respective gate of EpCAM-enriched samples because within the CellSearch cartridges, there are many leukocytes present in close proximity to each other, as shown in Figure 1, that are segmented as one object by the ACCEPT software. Therefore, the inclusion of such a parameter in the EpCAM-enriched samples would lead to an even higher underestimation of this population compared to the underestimation already introduced by cell clusters counted as one object. On the other hand, the removal of that parameter in case of blood samples with no enrichment (Figure 3), where it is very rare to find two or more leukocytes in close proximity, leads to the inclusion of artefacts and an overestimation of leukocytes.

For the acquisition of the fluorescence images of cells and EVs in blood without any pre-enrichment, the focus was set on four points distributed throughout each well to achieve optimal visualization of the cells, and the surface was afterwards automatically scanned. Since the objects were in suspension and not attached on a surface, most ldEVs and platelets, were out-of-focus with their blurring pattern influencing their perceived size, that seems much larger in the respective fluorescence images than it actually is. Hence, the size of ldEVs cannot be accurately derived using the immunofluorescence images (Figure 2). In case of EpCAM enriched samples, EVs are aligned on the same focal plane as the cells due to the design of the CellSearch magnets that result in a homogeneous cell distribution along the applied magnetic field [22]. Even in that case; however, the use of immunofluorescence images could lead to erroneous conclusions about the size of the EVs. That is even more profound when low magnification objectives are used in the fluorescence microscopes as in our case (10×/0.45 NA) limiting the determination of the size of EVs with confidence since each pixel of the acquired images corresponds to 0.64 × 0.64 μm^2^. More examples of correlated bright field and immunofluorescence images of ldEVs in whole blood samples could be found in Supplementary Figure S2.

### 3.4. Absolute and Relative Frequencies of Leukocytes and ldEVs in 7.5 mL of EpCA- Enriched Blood of Healthy Individuals and Metastatic Cancer Patients.

**Figure 4:**
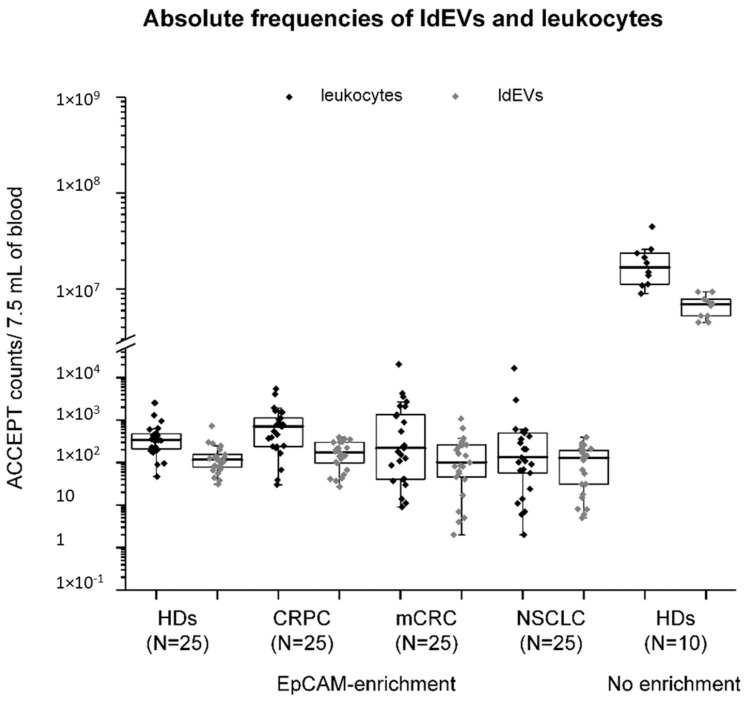
Absolute frequencies of leukocytes and large ldEVs in 7.5 mL of the blood of individuals with and without EpCAM enrichment. The interquartile range of the absolute frequencies of leukocytes (data in black dots) and ldEVs (data in grey dots) in whole blood of 10 healthy individuals and EpCAM enriched blood samples of 25 healthy individuals (HDs) and 75 EpCAM-enriched blood samples of metastatic prostate (CRPC), colorectal (mCRC) and non-small cell lung (NSCLC) cancer patients are shown in box plots. Whiskers indicate max and min values as estimated by Q3 + 1.5*IQR and Q1—1.5*IQR, respectively, where Q1: lower quartile, Q3: upper quartile and IQR: interquartile range. Each dot in the case of the blood of healthy individuals without EpCAM-enrichment corresponds to the mean values of 4–6 technical replicates.

The numbers of ldEVs and leukocytes in EpCAM-enriched 7.5 mL blood samples of 25 healthy individuals, 25 metastatic prostates, 25 colorectal, and 25 non-small cell lung cancer patients were determined and are presented in box plots (Figure 4). In addition, the number of ldEVs and leukocytes present in 0.02 μL of the blood of 10 healthy individuals with no enrichment was determined and extrapolated to 7.5 mL of blood for comparison (Figure 4). As it was expected, the leukocyte and ldEV frequencies are significantly depleted in the EpCAM-enriched blood samples of individuals, since EpCAM is an epithelial marker that is not expected to be expressed on the surface of leukocytes and ldEVs; therefore, leukocytes and ldEVs are not positively selected by the EpCAM ferrofluid. For each sample (with or without EpCAM enrichment), the relative frequencies of ldEVs over leukocytes were calculated. In the blood of healthy individuals with no enrichment, one ldEV was detected for every three leukocytes. In the EpCAM-enriched blood of both healthy individuals and metastatic cancer patients, the relative frequencies of ldEVs over leukocytes was found to be approximately half, with one ldEV being detected for every five (in case of samples from healthy individuals, prostate cancer, and non-small cell lung cancer patients) to six (in case of samples from colorectal cancer patients) leukocytes. The presence of ldEVs in higher relative frequencies in the the whole blood of individuals compared to EpCAM-enriched samples could be attributed to three reasons. Firstly, the blood samples are centrifuged at 800× *g* for 10 min, and the plasma fraction containing the majority of extracellular vesicles is not processed by the CellSearch system implying that half of the ldEVs detected in the blood samples without EpCAM enrichment end up in the plasma fraction. Secondly, the Fcγ receptors of leukocytes and ldEVs are expected to bind to the heavy chains rather than the antigen-binding sites of the antibodies against EpCAM that are conjugated to the ferrofluid. As ldEVs have fewer receptors due to their smaller surface, their carryover in the EpCAM-enriched sample should be lower than leukocytes. The third reason for that observation might be the lower CD45 signal of leukocytes and ldEVs in the images of the CellTracks Analyzer II compared to the imaging setup used in the case of the blood samples without EpCAM enrichment; the CellTracks Analyzer II uses a mercury arc lamp that results in a suboptimal excitation of the APC-conjugated antibody against CD45 in contrast to the other imaging setup that uses a light-emitting diode (LED) source. In combination with the smaller size of ldEVs, this could lead to ldEVs with a CD45 signal close to the background intensity not being considered as true events; thereby, underestimating the ldEV frequencies. In any case, the relative frequencies of ldEVs to leukocytes in blood samples with and without EpCAM enrichment are of a similar order of magnitude (1:3 and 1:5, respectively) supporting their pre-existence in the blood circulation of individuals and rejecting a possible hypothesis for their formation during the CellSearch procedure.

### 3.5. The Reproducibility of Measurements by Fluorescence Imaging and the Correlation with the Frequencies of Blood Cells by Hematology Analyzer

The technical variability (N = 4–6 technical replicates) of measuring cell populations in 0.02 μL of blood of healthy individuals without any pre-enrichment was assessed by performing 4–6 replicates of 0.02 μL blood from 10 healthy individuals. An average standard error of 25%, 18%, and 23% was obtained for leukocytes, red blood cells, and platelets, respectively. The respective standard error for ldEVs from the technical replicates was found to be 50% because of the very low frequency of ldEVs in 0.02 μL of blood processed, that was found to be 18 ± 5 (mean value ± SD). We expect that processing larger blood volumes would lead to lower technical variations.

**Figure 5:**
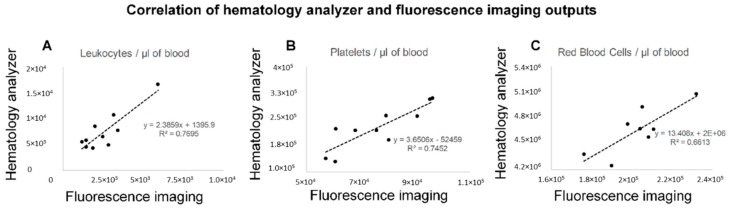
Scatter plots of leukocyte (Panel **A**), platelet (Panel **B**), and red blood cell frequencies (Panel **C**) in 1 μL of whole blood of 10 healthy individuals as estimated by fluorescence imaging and ACCEPT enumeration (*x*-axis) and by the hematology analyzer (*y*-axis). Correlation between the measurements of the two techniques was found as indicated by the R^2^. The mean counts of each population of 4–6 technical replicates were used in the case of the fluorescence imaging approach.

The averaged counts of the blood cell classes as estimated by the immunofluorescence imaging were extrapolated to 1 μL of blood and compared to the respective frequencies obtained by the hematology analyzer. The measurements of the fluorescence imaging were moderately correlated (R^2^ = 0.7) with the counts from the hematology analyzer, as shown in Figure 5. However, all cell populations were underestimated by the fluorescence imaging approach when compared to the hematology analyzer. That can be justified by the low CD45 expression of neutrophils that comprise 60–70% of the whole leukocyte population, the overlap and aggregation of red blood cells (Supplementary Figure S1) that are considered as one when enumerated using the ACCEPT software and the detection limit of fluorescence imaging in case of smaller size platelets.

## 4. Discussion

The Extracellular Vesicle field has focused so far on the biogenesis and functions of EVs with a size smaller than 1 μm secreted by various cells [26], including platelets [27], neutrophils [28], T and B lymphocytes [29], red blood cells [30], endothelial cells [31,32], and tumor cells [33]. However, the existing literature on the formation and frequencies of EVs larger than 1 μm in healthy individuals is very sparse, as they are considered to be apoptotic bodies, and as such not actively contributing in the intercellular communication. Nevertheless, recent findings in the cancer field shows the promising potential of large tdEVs as their load associated with clinical outcome in metastatic cancer patients [16,25] and their molecular cargo represents the mutational status of the tumor [9]. Our previous research on scanning electron microscopy imaging of CellSearch cartridges of castration-resistant prostate cancer patient samples after EpCAM enrichment [34] and the development of the open-source ACCEPT software for the automated enumeration of all fluorescently labeled objects from image data sets led to our first observations of the presence of DAPI-, CD45+, CK- objects [19]. We baptized these objects leukocyte-derived Extracellular Vesicles (ldEVs) and investigated their presence in digitally stored fluorescence images of CellSearch cartridges. The ldEVs had a similar size range to our previously reported DAPI-, CD45-, CK+ tumor-derived Extracellular Vesicles (tdEVs), that were detected after EpCAM enrichment in metastatic cancer patients [16,17]. Their detection raised questions regarding the pre-existence of these large ldEVs and tdEVs in the blood circulation of individuals or their formation as fragmentation by-products of leukocytes and CTCs, respectively during the CellSearch procedure.

Therefore, we decided to address the question of whether these EVs pre-exist in the blood circulation of individuals. Towards that direction, we enumerated ldEVs in blood samples of 10 healthy individuals without any pre-analytical or pre-enrichment steps and compared the frequencies of ldEVs and leukocytes in the whole blood to the frequency in EpCAM-enriched blood samples of 25 healthy individuals and 75 metastatic cancer patients. ldEVs and leukocytes were detected in a ratio of 1:3 in the blood of healthy individuals without any pre-enrichment and in 1:5 to 1:6 in the EpCAM-enriched blood of healthy individuals and metastatic cancer patients (Figure 4), supporting the pre-existence of these ldEVs in the blood circulation instead of their formation during the EpCAM enrichment. The lower relative frequencies of ldEVs to leukocytes in the EpCAM-enriched blood samples compared to the samples without EpCAM enrichment could be mainly explained by the blood fraction that is processed by the CellSearch system: blood samples are centrifuged at 800× *g* for 10 min, and the plasma fraction is discarded and not processed by the system. Using that centrifugation force, only EVs with a diameter above 1 μm will be in the blood fraction and will have the chance to come into contact with the EpCAM ferrofluid [35]. The measurements in the blood of healthy donors without EpCAM enrichment were reproducible, as confirmed by the standard deviations of the technical replicates, with a mean ± SD of 900 ± 254 ldEVs in 1 μL of the blood of healthy individuals. These results do not deviate a lot from the previously reported ldEV frequencies (median value: 356, interquartile range: 268–529) of Simak et al. in the plasma of healthy donors; ldEVs were larger than 200 nm and were defined as CD45+, CD105-, CD235a- by flow cytometry [36]. The use of solely one specific but weakly expressed inclusion marker, namely CD45 to define them using either approaches results in the underestimation of the whole circulating ldEV population; a point also stressed out by Lacroix et al. [37]. Further investigation of ldEVs in terms of their size distribution and surface marker expression using established techniques in the EV field, such as nanoparticle tracking analysis, electron microscopy, flow cytometry, and surface plasmon resonance imaging, would lead to their better profiling [10,38,39]. Eventually, a similar test to the hematology analyzer having as an output the EV populations (of platelet, erythrocyte, leukocyte, endothelial, and epithelial origin) in the biofluids of individuals could serve as an important diagnostic tool in clinical practice, since EVs have been associated with numerous pathophysiological conditions, such as thrombogenicity, inflammation, angiogenesis, and cancer [26,40,41,42,43,44].

Our study has several limitations. Although the extrapolated counts of leukocytes, platelets, and red blood cells per μL of blood correlated to the respective values of the hematology analyzer (Figure 5), the detected frequencies of all the cell populations were consistently lower as compared to the respective ones measured by the hematology analyzer. The particularly lower detection of red blood cells could be explained by the overlap and aggregation of more than one red blood cell segmented as one object by the open-source ACCEPT software and the large range in the distribution of the fluorescence intensity of CD235-APC in the fluorescence images (Supplementary Figure S1, Panel A) through which part of the red blood cells fall outside the applied gate. The underestimation of the leukocytes could be explained by the lower expression of CD45 by the granulocytes that consist to 60–70% of white blood cells. Τhe detection of the smaller platelet population and their secreted EVs is limited by our approach, because of the use of a 0.45 NA objective that results in a resolution of 0.64 μm/pixel. The abundance and high signal of the red blood cells prevented us from the detection and enumeration of low signal-to-background ratio red blood cell-derived EVs. Importantly, the Hoechst 33342 labeled nucleic acids in platelets could be detected by flow cytometry but not with our microscopy set-up. This observation implies that the zero DAPI signal detected with ACCEPT inside our previously reported tdEVs does not rule out the presence of nucleic acids within them. This is an important finding encouraging the further characterization of tdEVs that are immunomagnetically isolated based on their EpCAM expression [16,17,25]. That would come into agreement with the findings of Vagner et al. that the DNA cargo of large EVs reflect the genotype of prostate cancer patients [9]. The use of a membrane permeable dye, binding to both DNA and RNA, with a higher sensitivity, like SYTO13 [45] could also facilitate the detection of nucleic acids within the isolated EVs.

Interestingly, platelets have a similar size to ldEVs based on the immunofluorescence images of CD61 and CD45, respectively, with a minimum area of the detected objects being 9 μm^2^ based on our observations from the ACCEPT scatter plots. That area corresponds to a circular object of an approximate radius of 1.7 μm. However, it was not possible to confirm the size of ldEVs from the respective bright field images, because opposite to platelets, the contrast between the background and ldEV intensity was inadequate to detect them (Figure 2 and Supplementary Figure S2) suggesting that their physical properties (absorption coefficient, scattering coefficient, scattering anisotropy, refractive index) differ from the ones of platelets.

It is worth mentioning that ldEVs were found in 5–6 lower frequencies compared to leukocytes in EpCAM enriched samples, whereas tdEVs in our previous studies were detected in 10–20 times higher frequencies compared to CTCs [16]. That observation could be explained after taking into consideration some technical and biological facts. From a technical perspective, our study was limited by the resolution of a 10×/0.45 NA objective fluorescence microscope, implying that only the larger EVs with a larger than 1 μm diameter or the ones with a high expression of inclusion markers could be detected. CK is the inclusion marker used for the detection of tdEVs, whereas the detection of ldEVs is accomplished by the inclusion of CD45. Since the CK expression is intracellular and proportional to the volume instead of the surface, as in the case of the CD45 expression of ldEVs, CK is easier to detect in smaller tdEVs than CD45 in similar size ldEVs. Consequently, the CD45 expression may be present in more particles in blood samples but not exceeding the detection limit to be considered positive. Further characterization of the size distribution and the surface marker expression profile could elaborate on the detection limits of our technique. From a biological perspective, tdEVs could be found in higher frequencies either because of the increased apoptosis and fragmentation of CTCs in the blood circulation or because of different EV secretion pattern between normal and cancerous cells. Regarding the first hypothesis, the lifespan of neutrophils is around 24 h [46], whereas the circulation lifetime of CTCs has been estimated to be 1 to 2.4 h [47]; that; however, does not imply that CTCs are fragmented and cleared by the blood. On the contrary, *in vivo* animal studies showed the trap of more than 80% of viable CTCs by the liver and lung, that serve as “filter” organs, and the survival of CTCs for the prolonged time in a dormant state [48,49]. The survival of CTCs in the bloodstream is further supported by studies on their mechanical phenotype. Atomic force microscopy studies on cell lines suggest that cells with increased metastatic potential are more deformable (as expressed by Young’s modulus), compared to less metastatic or non-malignant cells [50,51]. These results were further confirmed in clinical samples from pleural effusions, where metastatic cells had lower stiffness compared to benign cells from the same effusions and leukocytes [52,53]. Interestingly, Sun et al. demonstrated that deformable cancer cells engulf neighboring ones with higher stiffness via entosis further encouraging the increased survivorship of CTCs [54]. Regarding the second hypothesis of different EV secretion pattern of CTCs and leukocytes, it is well known that cancer cells have reprogrammed metabolism and acquired traits that promote their survival and growth [55,56]. Recent findings of independent research groups converge into the survival of tumor cells regardless of their phenotypic characteristics of possible apoptosis, such as caspases activation, amoeboid phenotype and membrane blebbing [57,58]. Instead of undergoing apoptosis, cells with such traits have a more tumorigenic and invasive phenotype [59]. Hence, tumor cells may actively secrete EVs similar in size to apoptotic bodies, but without special receptors on their surface to be recognized and ingested by macrophages for their clearance as in case of healthy cells (e.g., white blood cells).

## 5. Conclusions

In conclusion, the relative frequencies of large (above 1 μm) leukocyte-derived Extracellular Vesicles (ldEVs) to leukocytes are similar in EpCAM-enriched blood samples of healthy individuals and cancer patients (1:6 to 1:5) as in the blood of healthy individuals without EpCAM enrichment (1:3), implying their pre-existence in the blood circulation rather than their formation from activated or apoptotic leukocytes using the CellSearch system. Furthermore, the Hoechst signal of platelets could not be detected using a similar fluorescence microscope as the CellTracks Analyzer II. These two findings have important implications for our previously reported tumor-derived Extracellular Vesicles (tdEVs), that were immunomagnetically co-isolated with CTCs based on their EpCAM expression from metastatic cancer patients [17]. Firstly, tdEVs are most likely not a result of CTC fragmentation during the CellSearch procedure and secondly, the presence of undetectable nucleic acids within ldEVs and tdEVs should not be excluded but instead further investigated. No conclusions could be drawn in regards to the smaller ldEV population, namely exosomes, since they are not expected to be detected with our imaging setup. Last but not least, our results do not allow for comparison of ldEVs between healthy individuals and cancer patients since the available image data sets of patients corresponded to only EpCAM enriched samples.

## Figures and Tables

**Table 1:** ACCEPT gates used for the automated enumeration of leukocytes, leukocyte-derived Extracellular Vesicles (ldEVs), platelets, and red blood cells in blood A with EpCAM enrichment and B without EpCAM enrichment.

		A. EpCAM Enrichment		B. No Enrichment	
**Leukocytes**	DAPI/Hoechst ^a^	Mean Intensity	>30	Mean Intensity	>30
		Max Intensity	>50	Max Intensity	>50
		Size	>16 μm^2^	Size	>16 μm^2^
	CD45	Mean Intensity	>30	Mean Intensity	>30
		Max Intensity	>50	Max Intensity	>50
				Size	≤400 μm^2^
	CK	Standard Deviation	≤5	n/a ^b^	
	CD61	n/a ^b^		Standard Deviation	≤5
	CD235a	n/a ^b^		Standard Deviation	≤5
	Extra channel	Standard Deviation	≤5	Standard Deviation	≤5
**ldEVs**	DAPI/Hoechst ^a^	Standard Deviation	≤5	Standard Deviation	≤5
	CD45	Mean Intensity	>30	Mean Intensity	>30
		Max Intensity	>50	Max Intensity	>50
		Perimeter	>5 pixels	Perimeter	>5 pixels
		Size	≤150	Size	≤150 μm^2^
		Eccentricity	≤0.85	Eccentricity	≤0.85
	CK	Standard Deviation	≤5	n/a ^b^	
	CD61	n/a ^b^		Standard Deviation	≤5
	CD235a	n/a ^b^		Standard Deviation	≤5
	Extra channel	Standard Deviation	≤5	Standard Deviation	≤5
**Platelets**	CD45	n/a ^b^		Standard Deviation	≤5
	CD61			Mean Intensity	>30
				Max Intensity	>50
				Perimeter	>5 pixels
				Size	≤150 μm^2^
				Eccentricity	≤0.85
	CD235a			Standard Deviation	≤5
				Standard Deviation	≤5
	Extra Channel			Standard Deviation	≤5
**Red blood cells**	Hoechst	n/a ^b^		Standard Deviation	≤5
	CD45			Standard Deviation	≤5
	CD61			Standard Deviation	≤5
	CD235a			Mean Intensity	>30
				Max Intensity	>50
				Perimeter	>5 pixels
	Extra Channel			Standard Deviation	≤5

^a^ DAPI was used in EpCAM-enriched blood and Hoechst in the blood without pre-enrichment, ^b^ n/a: not applicable.

## Supplementary Materials

The following are available online at https://www.mdpi.com/2073-4409/8/8/937/s1, Supplementary Figure S1: Examples of composite immunofluorescence images of blood samples without EpCAM enrichment, obtained with an inverted scanning fluorescence microscope using a 10×/0.45 NA (Panel A) and a 60×/0.7 NA objective (Panel B). Supplementary Figure S2: Examples of brightfield and immunofluorescence images of leukocyte- derived Extracellular Vesicles, enclosed within circles.
