# Supplementary material for: Leukocyte-Derived Extracellular Vesicles in Blood with and without EpCAM Enrichment
Source: Cells. 2019 Aug 20;8(8):937. doi: 10.3390/cells8080937 (PMC6721753; doi:10.3390/cells8080937)
Supplement: Supplementary file 1 [file cells-08-00937-s001.pdf]

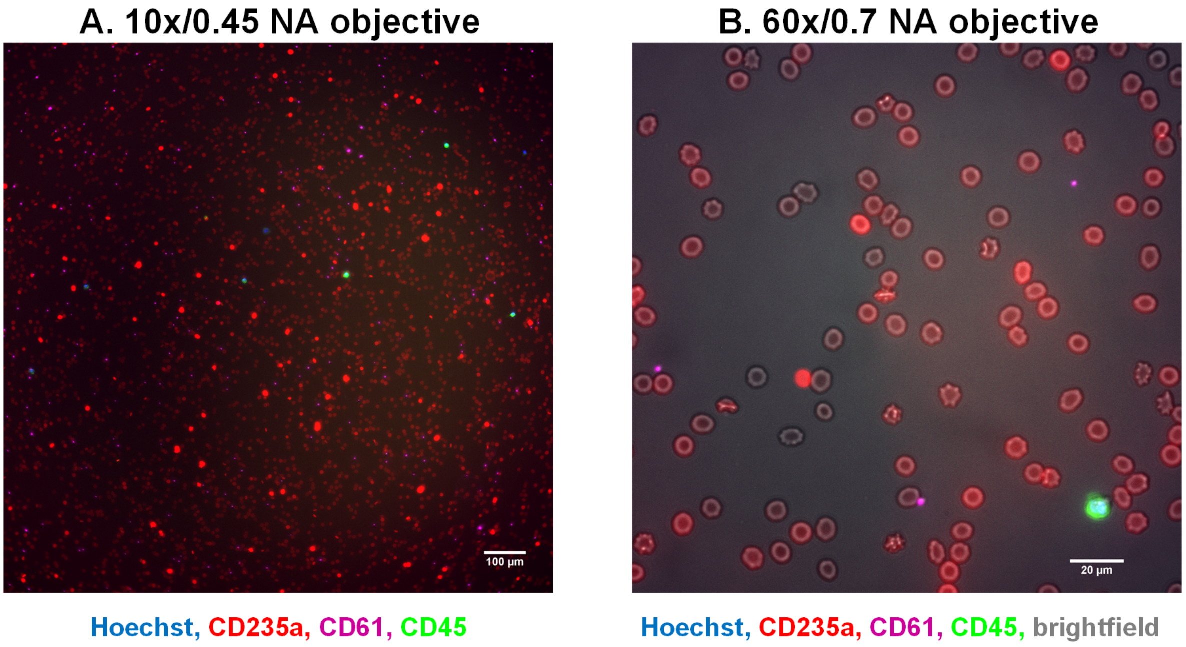

Supplementary Figure S1: Examples of composite immunofluorescence images of blood samples without EpCAM enrichment, obtained with an inverted scanning fluorescence microscope using a 10×/0.45 NA (Panel A) and a 60×/0.7 NA objective (Panel B). The images that were obtained with the 10× objective were used as an input in the open-source ACCEPT software for the automated enumeration of the different cell and EV populations.

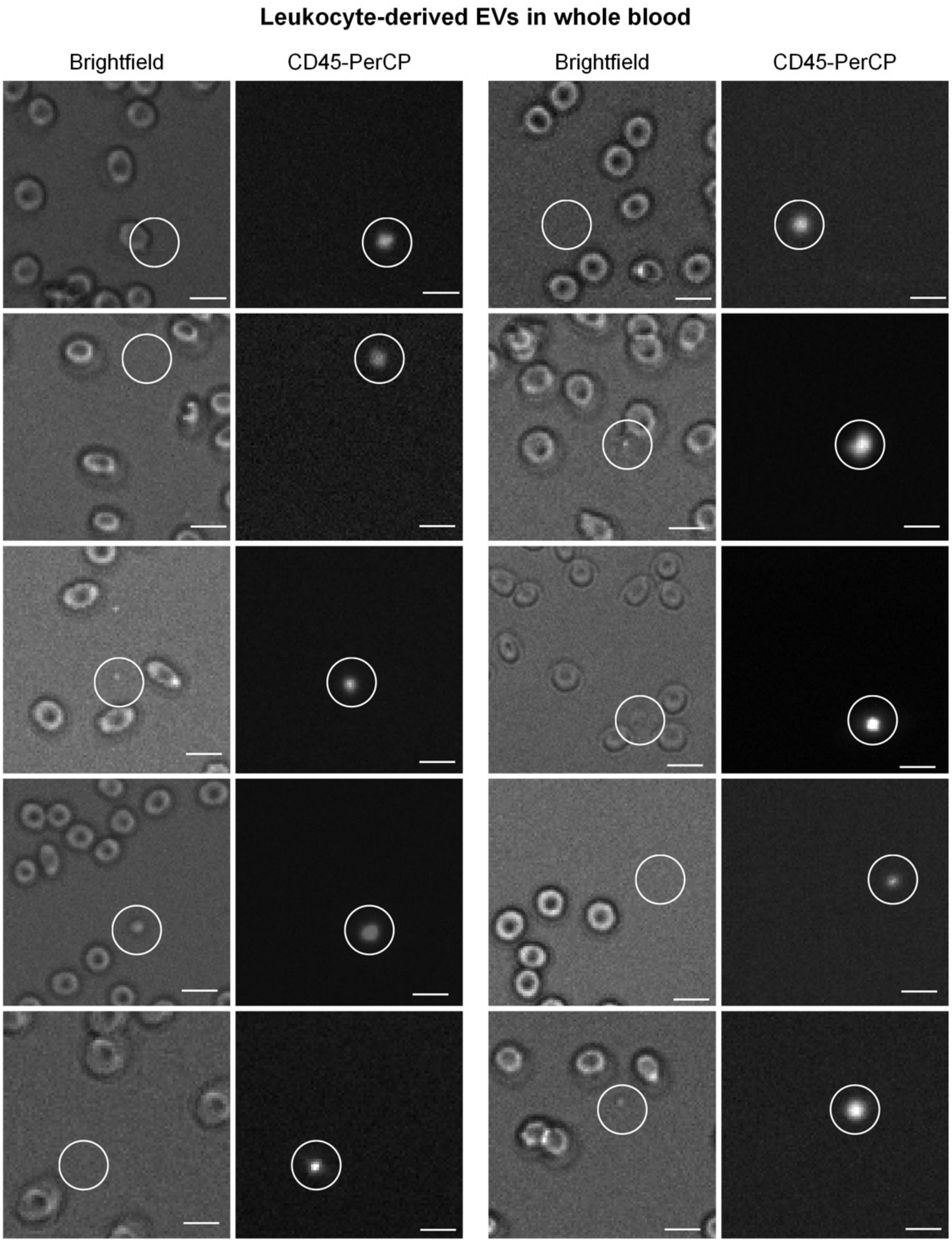

Supplementary Figure S2: Examples of brightfield and immunofluorescence images of leukocyte-derived Extracellular Vesicles, enclosed within circles. Scale bars indicate 10 μm.
